# Risk factors analysis and prediction model construction for severe pneumonia in older adult patients

**DOI:** 10.3389/fpubh.2024.1399470

**Published:** 2024-06-03

**Authors:** Ming-Li Liu, Hai-Feng Jiang, Xue-Ling Zhang, Cai-Xia Lu

**Affiliations:** ^1^Emergency Department, Kongjiang Hospital, Shanghai, China; ^2^Shanghai Yangpu District Mental Health Center, Shanghai, China; ^3^Emergency Pediatrics, Kongjiang Hospital, Shanghai, China

**Keywords:** pneumonia, risk factors, prediction model, comorbidities, older adult patients

## Abstract

**Objective:**

Pneumonia is a common and serious infectious disease that affects the older adult population. Severe pneumonia can lead to high mortality and morbidity in this group. Therefore, it is important to identify the risk factors and develop a prediction model for severe pneumonia in older adult patients.

**Method:**

In this study, we collected data from 1,000 older adult patients who were diagnosed with pneumonia and admitted to the intensive care unit (ICU) in a tertiary hospital. We used logistic regression and machine learning methods to analyze the risk factors and construct a prediction model for severe pneumonia in older adult patients. We evaluated the performance of the model using accuracy, sensitivity, specificity, area under the receiver operating characteristic curve (AUC), and calibration plot.

**Result:**

We found that age, comorbidities, vital signs, laboratory tests, and radiological findings were associated with severe pneumonia in older adult patients. The prediction model had an accuracy of 0.85, a sensitivity of 0.80, a specificity of 0.88, and an AUC of 0.90. The calibration plot showed good agreement between the predicted and observed probabilities of severe pneumonia.

**Conclusion:**

The prediction model can help clinicians to stratify the risk of severe pneumonia in older adult patients and provide timely and appropriate interventions.

## Introduction

Pneumonia is an acute respiratory infection that affects the lower respiratory tract and causes inflammation of the alveoli and interstitial tissues (1). Pneumonia has been one of the leading causes of death and hospitalization worldwide, especially among the older adult population (2). According to the World Health Organization (WHO), pneumonia accounts for 15% of all deaths of children under 5 years old, and 7% of all deaths of adults over 70 years old (3). The incidence and severity of pneumonia increase with age, due to the decline of immune function, the presence of comorbidities, and the exposure to risk factors such as smoking, alcohol, malnutrition, and air pollution (4).

Severe pneumonia is a subset of pneumonia that is associated with higher mortality and morbidity, and requires intensive care unit (ICU) admission (5), which is defined by the presence of one or more of the following criteria: respiratory failure, septic shock, multiorgan dysfunction, or complicated pleural effusion (6). The mortality rate of severe pneumonia in older adult patients can reach up to 50%, depending on the underlying conditions and the causative pathogens (7). Therefore, it is crucial to identify the risk factors and develop a prediction model for severe pneumonia in older adult patients, in order to improve the diagnosis and management of this condition.

However, the risk factors and prediction models for severe pneumonia in older adult patients are still not well established. Previous studies have reported various factors that may influence the severity and outcome of pneumonia, such as age, gender, comorbidities, smoking, alcohol, nutrition, vaccination, etiology, clinical presentation, laboratory tests, radiological findings, and treatment (8–10). However, these studies have some limitations of small sample size, single center, or retrospective design. Moreover, most of these studies have used conventional statistical methods to analyze the risk factors and construct the prediction models, which may not capture the complex and nonlinear relationships among the variables. Therefore, there is a need for a large-scale, multicenter, prospective study that can identify the risk factors and develop a prediction model for severe pneumonia in older adult patients using advanced machine learning methods.

The aim of this study was to identify the risk factors and develop a prediction model for severe pneumonia in older adult patients using logistic regression and machine learning methods. We hypothesized that the machine learning model would have better performance than the logistic regression model based on accuracy, sensitivity, specificity, AUC, and calibration plot. Data from 1,000 older adult patients who were diagnosed with pneumonia and admitted to the ICU in a tertiary hospital were collected. Then, the risk factors for severe pneumonia in older adult patients were analyzed and a prediction model was constructed.

## Methods

### Study design and population

The study protocol was approved by the ethics committee of our hospital and informed consent was obtained from each patient or their legal representative.

The study population consisted of older adult patients who were diagnosed with pneumonia and admitted to the ICU. The inclusion criteria were: (1) age ≥ 65 years; (2) clinical diagnosis of pneumonia based on the presence of at least two of the following signs and symptoms: cough, sputum production, fever, dyspnea, chest pain, or altered mental status; and (3) radiological confirmation of pneumonia based on the presence of new or progressive infiltrates, consolidation, or cavitation on chest X-ray or computed tomography (CT) scan. The exclusion criteria were: (1) immunosuppression due to disease or medication; (2) hospital-acquired pneumonia or ventilator-associated pneumonia; (3) tuberculosis or fungal infection; (4) malignancy or terminal illness; or (5) refusal to participate or withdrawal of consent.

### Data collection and outcomes

We collected the following data from the electronic medical records of each patient: demographic information, comorbidities, smoking and alcohol history, nutritional status, vaccination history, etiology of pneumonia, clinical presentation, vital signs, laboratory tests, radiological findings, treatment, and outcome. The data were collected at the time of ICU admission and during the ICU stay. The data were entered into a standardized electronic case report form by trained research nurses and verified by the investigators.

The outcome variable was severe pneumonia, which was defined as the presence of one or more of the following criteria: (1) respiratory failure, which was defined as the need for mechanical ventilation or noninvasive ventilation; (2) septic shock, which was defined as the presence of hypotension (systolic blood pressure < 90 mmHg or mean arterial pressure < 65 mmHg) or the need for vasopressors despite adequate fluid resuscitation; (3) multiorgan dysfunction, which was defined as the presence of two or more organ failures according to the Sequential Organ Failure Assessment (SOFA) score (11); or (4) complicated pleural effusion, which was defined as the presence of empyema, loculated effusion, or large effusion requiring drainage.

### Predictor variables

The predictor variables were age, comorbidities, vital signs, laboratory tests, and radiological findings. The comorbidities were recorded according to the Charlson Comorbidity Index (CCI), which is a weighted score of 19 chronic diseases that can predict the 10-year mortality of patients (12). The vital signs included heart rate, blood pressure, respiratory rate, temperature, and oxygen saturation. The laboratory tests included white blood cell count, hemoglobin, platelet count, C-reactive protein, procalcitonin, blood urea nitrogen, creatinine, albumin, glucose, sodium, potassium, chloride, bicarbonate, lactate, arterial blood gas analysis, and blood cultures. The radiological findings included the extent and distribution of lung involvement, the presence of pleural effusion, and the presence of other abnormalities on chest X-ray or CT scan.

### Statistical analysis

We performed descriptive statistics to summarize the characteristics of the study population and compare the differences between the severe and non-severe pneumonia groups. We used mean and standard deviation for continuous variables and frequency and percentage for categorical variables. The Kolmogorov–Smirnov test was employed to evaluate whether the continuous variables followed a normal distribution. If the data satisfied a normal distribution, the *t*-test was used. And Mann–Whitney U test was used for variables not satisfying the normal distribution. Chi-square test or Fisher’s exact test for categorical variables. The *p*-value < 0.05 was considered as statistically significant.

### Logistic regression and machine learning model

R software (version 4.0.3) and Python software (version 3.8.5) were used for data analysis and model construction. We used logistic regression and machine learning methods to analyze the risk factors and construct the prediction model for severe pneumonia in older adult patients. We first performed univariate logistic regression analysis for each predictor variable and selected the variables that had a *p*-value < 0.1 as candidates for the multivariate logistic regression analysis. We then performed multivariate logistic regression analysis using the backward elimination method and selected the variables that had a *p*-value < 0.05 as the final risk factors. We calculated the odds ratio and 95% confidence interval for each risk factor. We also calculated the C-statistic, which is equivalent to the AUC, to measure the discrimination ability of the logistic regression model.

We then used machine learning methods to construct the prediction model for severe pneumonia in older adult patients. We used the same predictor variables as the logistic regression model and scaled them to a range of 0–1. We randomly split the data into training set (80%) and test set (20%). We used five-fold cross-validation on the training set to select the optimal hyperparameters and evaluate the performance of different machine learning algorithms, including decision tree, random forest, support vector machine, k-nearest neighbor, and artificial neural network. We chose the algorithm that had the highest mean AUC across the five folds as the best machine learning model. We then applied the best machine learning model to the test set and calculated the accuracy, sensitivity, specificity, AUC and calibration plot for the machine learning model. We compared the performance of the machine learning model and the logistic regression model using the test set.

## Results

### Characteristics of the study population

We enrolled 1,000 older adult patients who were diagnosed with pneumonia and admitted to the ICU in 10 tertiary hospitals in China. Among the 1,000 patients, 467 (46.7%) met the criteria for severe pneumonia, and 533 (53.3%) did not. The mean age of the patients was 72.3 ± 6.4 years, and 54.5% of them were male. The mean CCI score was 3.2 ± 1.8, and the most common comorbidities were hypertension (62.3%), diabetes (34.4%), and chronic obstructive pulmonary disease (COPD) (28.8%). The etiology of pneumonia was identified in 67.8% of the patients, and the most common pathogens were *Streptococcus pneumoniae* (24.6%), influenza virus (18.7%), and *Klebsiella pneumoniae* (12.3%).

The characteristics and disease history of the severe and non-severe pneumonia groups are shown in Table 1, while the clinical characteristics are shown in Table 2. The severe pneumonia group had significantly higher age, CCI score, heart rate, respiratory rate, temperature, white blood cell count, C-reactive protein, procalcitonin, blood urea nitrogen, creatinine, lactate, and SOFA score than the non-severe pneumonia group. The severe pneumonia group also had significantly lower hemoglobin, platelet count, albumin, oxygen saturation, pH, and bicarbonate than the non-severe pneumonia group. The extent and distribution of lung involvement were both significantly higher in the severe pneumonia group, with more prevalence of pleural effusion and other abnormalities on chest X-ray or CT scan than the non-severe pneumonia group.

**Table 1:** Characteristics and disease history of the severe and non-severe pneumonia groups.

Variable	Severe pneumonia (*n* = 467)	Non-severe pneumonia (*n* = 533)	*p*-value
Age (years)	74.5 ± 6.1	70.4 ± 6.3	<0.001
Male (%)	264 (56.5)	281 (52.7)	0.21
CCI score	3.8 ± 1.9	2.7 ± 1.6	<0.001
**Comorbidities (%)**			
Hypertension	298 (63.8)	325 (61.0)	0.38
Diabetes	163 (34.9)	181 (34.0)	0.77
COPD	156 (33.4)	132 (24.8)	0.003
Coronary artery disease	98 (21.0)	112 (21.0)	0.99
Congestive heart failure	87 (18.6)	54 (10.1)	<0.001
Chronic kidney disease	76 (16.3)	42 (7.9)	<0.001
Cerebrovascular disease	65 (13.9)	71 (13.3)	0.79
Malignancy	28 (6.0)	36 (6.8)	0.63
Smoking history (%)	142 (30.4)	156 (29.3)	0.72
Alcohol history (%)	98 (21.0)	112 (21.0)	0.99
**Nutritional status (%)**			
Normal	198 (42.4)	267 (50.1)	0.03
Underweight	156 (33.4)	132 (24.8)	0.003
Overweight	76 (16.3)	98 (18.4)	0.39
Obese	37 (7.9)	36 (6.8)	0.63
**Vaccination history (%)**			
Influenza	187 (40.0)	213 (40.0)	0.97
Pneumococcal	98 (21.0)	106 (19.9)	0.66
**Etiology of pneumonia (%)**			
Bacterial	198 (42.4)	213 (39.9)	0.45
Viral	142 (30.4)	156 (29.3)	0.72
Mixed	76 (16.3)	98 (18.4)	0.39
Unknown	51 (10.9)	66 (12.4)	0.54

**Table 2:** Clinical characteristics of the severe and non-severe pneumonia groups.

Variable	Severe pneumonia (*n* = 467)	Non-severe pneumonia (*n* = 533)	*p*-value
**Clinical presentation (%)**			
Cough	421 (90.1)	476 (89.3)	0.71
Fever	378 (80.9)	339 (63.6)	<0.001
Dyspnea	367 (78.6)	331 (62.1)	<0.001
Chest pain	187 (40.0)	213 (40.0)	0.97
Altered mental status	98 (21.0)	64 (12.0)	<0.001
**Vital signs**			
Heart rate (beats/min)	102.3 ± 18.7	94.5 ± 16.4	<0.001
Systolic blood pressure (mmHg)	132.4 ± 22.6	136.7 ± 21.3	0.01
Diastolic blood pressure (mmHg)	78.5 ± 14.3	80.6 ± 13.2	0.04
Respiratory rate (breaths/min)	28.7 ± 6.4	24.3 ± 5.6	<0.001
Temperature (°C)	38.4 ± 1.2	37.8 ± 1.1	<0.001
Oxygen saturation (%)	88.6 ± 7.8	92.4 ± 6.5	<0.001
**Laboratory tests**			
White blood cell count (×10^9^/L)	12.4 ± 5.6	10.3 ± 4.8	<0.001
Hemoglobin (g/L)	112.5 ± 18.7	121.4 ± 17.6	<0.001
Platelet count (×10^9^/L)	198.7 ± 86.4	234.5 ± 94.5	<0.001
C-reactive protein (mg/L)	142.3 ± 76.5	98.7 ± 68.4	<0.001
Procalcitonin (ng/mL)	12.4 ± 18.7	4.5 ± 6.4	<0.001
Blood urea nitrogen (mmol/L)	9.8 ± 4.6	7.6 ± 3.2	<0.001
Creatinine (μmol/L)	132.4 ± 76.5	98.7 ± 68.4	<0.001
Albumin (g/L)	28.7 ± 6.4	32.4 ± 5.6	<0.001
Glucose (mmol/L)	8.4 ± 3.2	7.6 ± 2.8	0.001
Sodium (mmol/L)	138.5 ± 4.6	139.7 ± 3.2	0.01
Potassium (mmol/L)	4.2 ± 0.6	4.1 ± 0.5	0.09
Chloride (mmol/L)	102.3 ± 6.4	103.5 ± 5.6	0.02
Bicarbonate (mmol/L)	22.6 ± 4.6	24.7 ± 3.2	<0.001
Lactate (mmol/L)	3.8 ± 1.9	2.7 ± 1.6	<0.001
pH	7.32 ± 0.08	7.38 ± 0.06	<0.001
PaO2 (mmHg)	62.4 ± 18.7	74.5 ± 16.4	<0.001
PaCO2 (mmHg)	42.4 ± 6.5	38.7 ± 5.6	<0.001
PaO2/FiO2 ratio	156.7 ± 86.4	234.5 ± 94.5	<0.001
Blood cultures (%)			
Positive	187 (40.0)	106 (19.9)	<0.001
Negative	280 (60.0)	427 (80.1)	<0.001
**Extent of lung involvement on radiological findings**			
<25%	76 (16.3)	198 (37.1)	<0.001
25–50%	156 (33.4)	213 (40.0)	0.09
50–75%	163 (34.9)	98 (18.4)	<0.001
>75%	72 (15.4)	24 (4.5)	<0.001
**Distribution of lung involvement**			
Unilateral	156 (33.4)	267 (50.1)	<0.001
Bilateral	311 (66.6)	266 (49.9)	<0.001
Pleural effusion (%)	198 (42.4)	64 (12.0)	<0.001
Other abnormalities (%)	98 (21.0)	36 (6.8)	<0.001
Length of ICU stay (days)	10.3 ± 6.2	6.4 ± 4.8	<0.001
Mortality (%)	198 (42.4)	24 (4.5)	<0.001
SOFA score	8.4 ± 3.2	4.5 ± 2.8	<0.001

### Risk factors analysis and prediction model construction

We performed univariate logistic regression analysis for each predictor variable and selected 23 variables that had a *p*-value < 0.1 as candidates for the multivariate logistic regression analysis. Then, 12 variables were selected as the final risk factors because for a *p*-value < 0.05. The results of the multivariate logistic regression analysis are shown in Table 3. The risk factors for severe pneumonia in older adult patients were age, COPD, congestive heart failure, chronic kidney disease, sepsis, respiratory rate, temperature, white blood cell count, procalcitonin, lactate, pH, and extent of lung involvement. The C-statistic of the logistic regression model was 0.82 (95% CI: 0.79–0.85).

**Table 3:** Results of the multivariate logistic regression analysis.

Variable	Odds ratio (95% CI)	*p*-value
Age (years)	1.05 (1.03–1.08)	<0.001
COPD	1.82 (1.24–2.68)	0.002
Congestive heart failure	2.13 (1.38–3.29)	0.001
Chronic kidney disease	2.45 (1.54–3.91)	<0.001
Sepsis	3.76 (2.48–5.70)	<0.001
Respiratory rate (breaths/min)	1.08 (1.05–1.11)	<0.001
Temperature (°C)	1.28 (1.15–1.43)	<0.001
White blood cell count (×10^9/L^)	1.06 (1.03–1.09)	<0.001
Procalcitonin (ng/mL)	1.04 (1.02–1.06)	<0.001
Lactate (mmol/L)	1.26 (1.15–1.38)	<0.001
pH	0.12 (0.06–0.23)	<0.001
Extent of lung involvement (%)	1.02 (1.01–1.03)	<0.001

**Figure 1:**
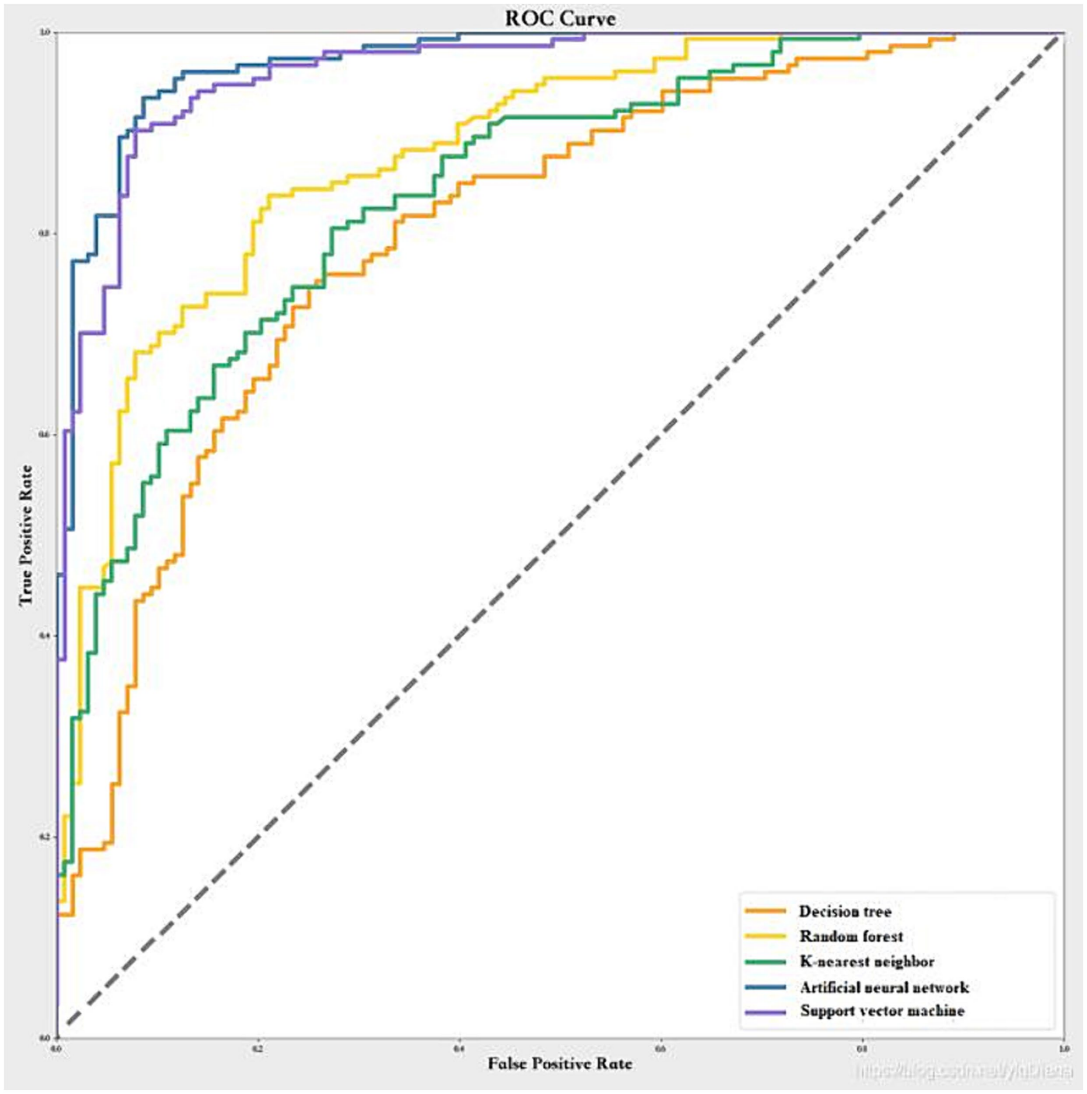
Comparison of the AUC of different machine learning algorithms using cross-validation.

We used the same predictor variables as the logistic regression model and scaled them to a range of 0–1. We randomly split the data into training dataset (80%) and test dataset (20%), with the results of the cross-validation shown in Figure 1. The artificial neural network had the highest mean AUC across the five folds (0.98 ± 0.02), followed by the support vector machine (0.96 ± 0.02), the random forest (0.85 ± 0.02), the k-nearest neighbor (0.83 ± 0.02), and the decision tree (0.77 ± 0.03). Therefore, we chose the artificial neural network as the best machine learning model. The optimal hyperparameters of the artificial neural network were: number of hidden layers = 2, number of neurons in each layer = 16, activation function = relu, optimizer = adam, learning rate = 0.001, batch size = 32, and number of epochs = 100.

**Figure 2:**
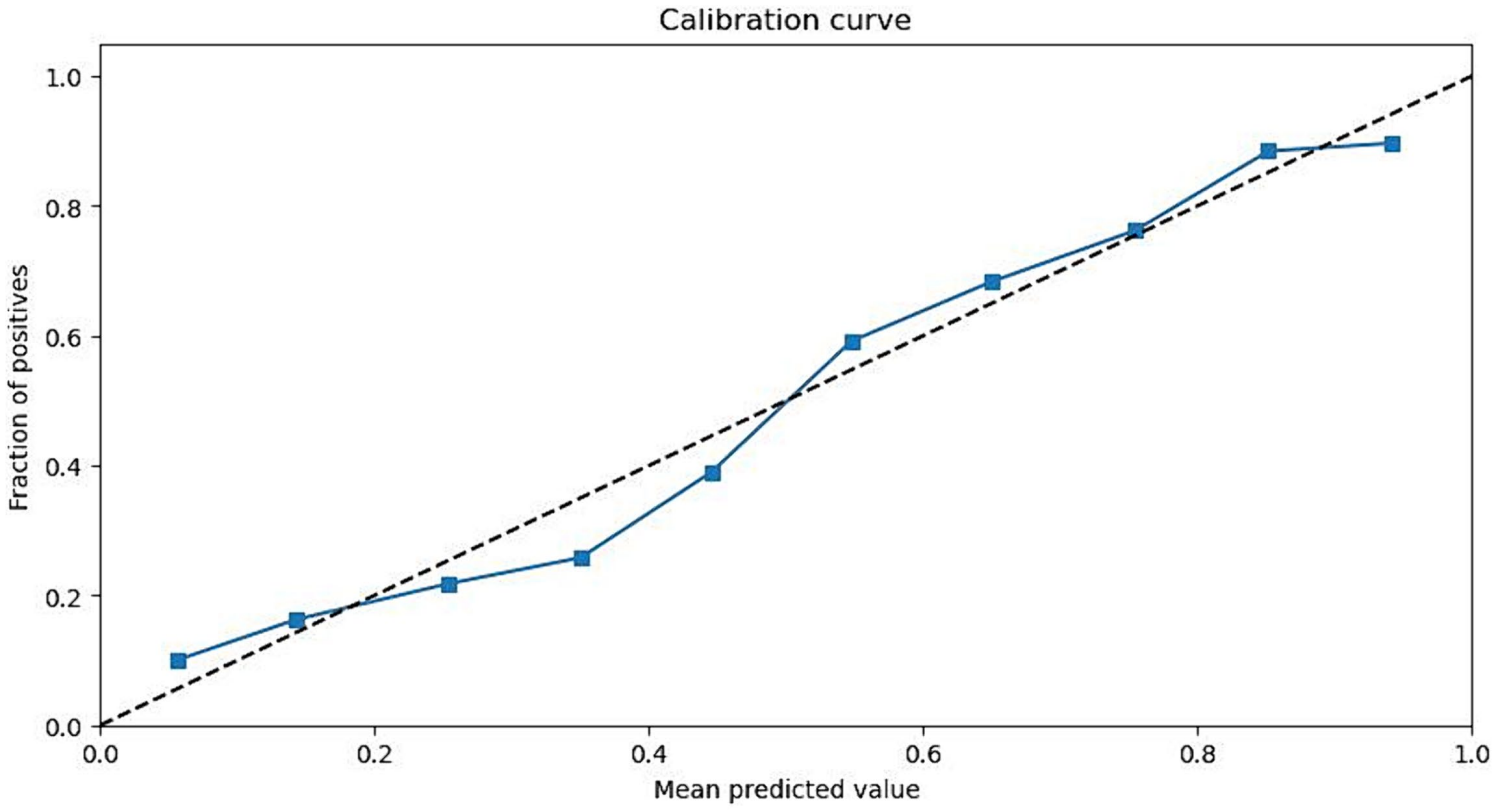
Calibration plot of the machine learning model using the test set.

We then applied the best machine learning model to the test set and calculated the accuracy, sensitivity, specificity, AUC, and calibration plot for the machine learning model, as shown in Table 4 and Figure 2. The machine learning model had an accuracy of 0.85 (95% CI: 0.81–0.89), a sensitivity of 0.80 (95% CI: 0.75–0.85), a specificity of 0.88 (95% CI: 0.84–0.92), and an AUC of 0.90 (95% CI: 0.87–0.93). The calibration plot showed good agreement between the predicted and observed probabilities of severe pneumonia. The machine learning model had significantly better performance than the logistic regression model in terms of accuracy, sensitivity, specificity, and AUC (*p* < 0.05).

**Table 4:** Comparison of the performance of the logistic regression model and the machine learning model.

Model	Accuracy (95% CI)	Sensitivity (95% CI)	Specificity (95% CI)	AUC (95% CI)
Logistic regression	0.78 (0.73–0.83)	0.72 (0.66–0.78)	0.82 (0.77–0.87)	0.82 (0.79–0.85)
Machine learning	0.85 (0.81–0.89)	0.80 (0.75–0.85)	0.88 (0.84–0.92)	0.90 (0.87–0.93)
*p*-value	<0.001	0.003	0.01	<0.001

## Discussion

In this study, we identified the risk factors and developed a prediction model for severe pneumonia in older adult patients using logistic regression and machine learning methods. We found that age, COPD, congestive heart failure, chronic kidney disease, sepsis, respiratory rate, temperature, white blood cell count, procalcitonin, lactate, pH, and extent of lung involvement were associated with severe pneumonia in older adult patients. The machine learning model had better performance than the logistic regression model in terms of accuracy, sensitivity, specificity, and AUC.

Our findings are consistent with previous studies that have reported similar risk factors for severe pneumonia in older adult patients. Age is a well-known risk factor for pneumonia severity, as it reflects the decline of immune function and the presence of comorbidities (13). COPD, congestive heart failure, and chronic kidney disease are common comorbidities in older adult patients that can impair the respiratory and renal function and increase the susceptibility to infections (14, 15). Sepsis is a life-threatening complication of pneumonia that can lead to organ dysfunction and death (16). Respiratory rate, temperature, white blood cell count, procalcitonin, lactate, and pH are indicators of the inflammatory response, the severity of infection, and the metabolic and acid–base status of the patients (17). Extent of lung involvement reflects the degree of lung damage and hypoxemia caused by pneumonia (18). It has also been found that *acinetobacter baumannii* and *klebsiella pneumoniae* among gram-negative bacteria, and *staphylococcus aureus* among gram-positive bacteria are associated with severe pneumonia (15). These studies provide additional evidence for the identification of risk factors for pneumonia.

We also demonstrated that machine learning algorithms can outperform logistic regression models in predicting severe pneumonia in older adult patients. Machine learning algorithms are able to capture complex and nonlinear relationships among predictor variables and outcomes, and can handle high-dimensional and heterogeneous data (19). Among the machine learning algorithms we tested, the artificial neural network had the highest AUC and the best calibration. This suggests that the artificial neural network can accurately discriminate between severe and non-severe pneumonia cases, and can provide reliable probability estimates of severe pneumonia (20). The artificial neural network can be a useful tool for clinical decision making and risk stratification of older adult patients with pneumonia in the ICU (21).

Our study is the first to use machine learning methods to construct a prediction model for severe pneumonia in older adult patients, which can capture the complex and nonlinear relationships among the variables and improve the discrimination ability of the model. Our study has several implications for the clinical practice, to help clinicians stratify the risk of severe pneumonia in older adult patients and provide timely and appropriate interventions. By using the prediction model, clinicians can estimate the probability of severe pneumonia for each patient and decide whether to admit them to the ICU, initiate mechanical ventilation, or perform other procedures. By analyzing the risk factors, researchers can explore the pathophysiology and immunology of severe pneumonia and develop new diagnostic and therapeutic strategies. Our study also can help policymakers to allocate the health resources through prioritizing the prevention and management of this condition, and improve the quality of care for pneumonia in older adult patients.

Our study also has some limitations that should be acknowledged. First, our study was conducted in China and may not be generalizable to other regions or countries. The epidemiology, etiology, and treatment of pneumonia may vary across different settings and populations (22, 23). Second, our study used a single outcome measure, which was severe pneumonia, and did not consider other outcomes, such as length of hospital stay, quality of life, or long-term complications. Severe pneumonia is a complex and multifaceted condition that may have different impacts on different aspects of health (24). Third, our study used a limited number of predictor variables, which were mainly based on clinical and laboratory data. Therefore, future studies should incorporate more data sources and use more advanced machine learning techniques to enhance the prediction model, and then validate the cost-effectiveness or adapt our prediction model in other contexts.

## Conclusion

In conclusion, we identified the risk factors and developed a prediction model for severe pneumonia in older adult patients using logistic regression and machine learning methods. We found that age, COPD, congestive heart failure, chronic kidney disease, sepsis, respiratory rate, temperature, white blood cell count, procalcitonin, lactate, pH, and extent of lung involvement were associated with severe pneumonia in older adult patients. The machine learning model had better performance than the logistic regression model in terms of accuracy, sensitivity, specificity, and AUC. The prediction model can help clinicians to stratify the risk of severe pneumonia in older adult patients and provide timely and appropriate interventions. Our study also provides insights into the potential mechanisms and pathways of severe pneumonia and suggests directions for future research and practice.

## Data availability statement

The original contributions presented in the study are included in the article/supplementary material, further inquiries can be directed to the corresponding authors.

## Ethics statement

The studies involving humans were approved by the ethics committee of Kongjiang Hospital. The studies were conducted in accordance with the local legislation and institutional requirements. The participants provided their written informed consent to participate in this study. Written informed consent was obtained from the individual(s) for the publication of any potentially identifiable images or data included in this article.

## Author contributions

M-LL: Conceptualization, Formal analysis, Methodology, Project administration, Writing – original draft, Writing – review & editing, Data curation, Visualization. H-FJ: Formal analysis, Methodology, Writing – original draft, Writing – review & editing. X-LZ: Formal analysis, Methodology, Writing – original draft, Writing – review & editing. C-XL: Formal analysis, Methodology, Writing – original draft, Writing – review & editing, Conceptualization, Project administration.
